# Japanese Herbal Medicine Ninjinyoeito Mediates Its Orexigenic Properties Partially by Activating Orexin 1 Receptors

**DOI:** 10.3389/fnut.2020.00005

**Published:** 2020-02-27

**Authors:** Kanako Miyano, Kaori Ohshima, Nozomi Suzuki, Saho Furuya, Yuki Yoshida, Miki Nonaka, Yoshikazu Higami, Kazumi Yoshizawa, Hideaki Fujii, Yasuhito Uezono

**Affiliations:** ^1^Division of Cancer Pathophysiology, National Cancer Center Research Institute, Tokyo, Japan; ^2^Laboratory of Pharmacology and Therapeutics, Faculty of Pharmaceutical Sciences, Tokyo University of Science, Chiba, Japan; ^3^Department of Medicinal Chemistry, School of Pharmacy, Kitasato University, Tokyo, Japan; ^4^Laboratory of Molecular Pathology and Metabolic Disease, Faculty of Pharmaceutical Sciences, Tokyo University of Science, Chiba, Japan; ^5^Division of Supportive Care Research, National Cancer Center Exploratory Oncology Research and Clinical Trial Center, Tokyo, Japan; ^6^Innovation Center for Supportive, Palliative and Psychosocial Care, National Cancer Center Hospital, Tokyo, Japan

**Keywords:** anorexia, *citrus unshiu* peel, kampo medicine, ninjinyoeito, orexin 1 receptor

## Abstract

Cancer cachexia is highly prevalent in patients with progressive cancer and is characterized by decreased food consumption, and body weight. Japanese herbal medicine Ninjinyoeito (NYT), composed of 12 herbal crude drugs, is prescribed in Asian countries to improve several symptoms such as anorexia and fatigue, which are commonly observed in patients with cancer cachexia. However, the action mechanisms of NYT in improving anorexia or fatigue in patients with cancer are not clear. Therefore, in the present study, we examined the effects of NYT on the activities of several G-protein-coupled receptors (GPCRs), which activate hyperphagia signaling in the central nervous system, using an *in vitro* assay with the CellKey™ system, which detects the activation of GPCRs as a change in intracellular impedance (ΔZ). NYT increased the ΔZ of human embryonic kidney 293 (HEK293) cells expressing orexin 1 receptor (OX1R) and those expressing neuropeptide Y1 receptor (NPY1R) in a dose-dependent manner. On the contrary, NYT did not significantly increase the ΔZ of HEK293A cells expressing growth hormone secretagogue receptor (GHSR) and those expressing NPY5R. The selective OX1R antagonist SB674042 significantly decreased the NYT-induced increase in ΔZ in OX1R-expressing cells. Contrarily, the selective NPY1R antagonist BIBO3340 failed to inhibit the NPY-induced increase in ΔZ in NPY1R-expressing cells. Additionally, we prepared modified NYT excluding each one of the 12 herbal crude drugs in NYT and investigated the effects on the activity of OX1R. Among the 12 modified NYT formulations, the one without *citrus unshiu* peel failed to activate OX1R. A screening of each of the 12 herbal crude drugs showed that *citrus unshiu* peel significantly activated OX1R, which was significantly suppressed by SB674042. These finding suggest that NYT and *citrus unshiu* peel could increase food intake via activation of orexigenic OX1R-expressing neurons in the hypothalamus. This study provides scientific evidence to support the potential of NYT for cancer patients with anorexia.

## Introduction

Cancer cachexia, which is characterized by a decrease in body weight and food consumption, occurs in 80% of patients with progressive cancer, causing at least 20% of cancer-related deaths (1–3). This syndrome not only decreases the quality of life (QOL) but also attenuates the efficacy of chemotherapy (4–7). Studies suggest that cancer cachexia is caused by complicated interrelation among several mediators in the hypothalamus, such as hormones (e.g., leptin and ghrelin), and neuropeptides (e.g., neuropeptide Y and orexin), which regulate food intake (8–10). However, the mechanisms underlying this syndrome are not fully understood, and appropriate therapies for the treatment of cancer cachexia have not been established. The current treatment options for cancer cachexia are far from being satisfactory because of the lack of effective drugs currently available (6).

Ninjinyoeito (NYT), a traditional Japanese kampo medicine that contains extracted ingredients of 12 herbal crude drugs, is approved by Japan's Ministry of Health, Labor, and Welfare as a prescribed medicine in clinical practice. Since the 16th century, NYT has been prescribed in Japan and other Asian countries to ameliorate diseases and improve several symptoms such as anorexia and fatigue (11). In addition, several studies have shown that some herbal crude drugs of NYT improved appetite in a cancer cachexia model of animals or patients with cancer (12–16). However, the action mechanisms of NYT in improving anorexia and/or fatigue in cancer cachexia–anorexia syndrome are not clear.

Therefore, in the present study, we examined the effects of NYT on the activities of several G-protein-coupled receptors (GPCRs), which activate hyperphagia signaling in the central nervous system (CNS). With respect to GPCR-activating hyperphagia signaling, we focused on activated appetite-stimulating receptors, such as growth hormone secretagogue receptor 1a (GHSR), neuropeptide Y1 receptor (NPY1R), neuropeptide Y5 receptor (NPY5R), and orexin 1 receptor (OX1R) (17–32). First, we analyzed the effects of NYT on the activities of these receptors. Second, we identified active medicinal herbs contained in NYT by screening both modified NYT excluding 1 of the 12 herbal crude drugs and each of the 12 herbal crude drugs contained in NYT.

## Materials and Methods

### Chemicals and Reagents

The following reagents and medium were used in the present study: poly-D-lysine and bovine serum albumin (BSA) (Sigma-Aldrich, St. Louis, MO, USA); fetal bovine serum (FBS), Dulbecco's modified Eagle's medium (DMEM), and geneticin (Gibco, Carlsbad, CA, USA); penicillin/streptomycin and 4-(2-hydroxyethyl)-1-piperazineethanesulfonic acid (HEPES) (Nacalai Tesque, Kyoto, Japan); and DMEM (Fujifilm Wako Pure Chemical, Osaka, Japan).

NYT extract powder (lot no. 15112017), the base powder of NYT without excipients, was obtained from Kracie Pharma, Ltd. (Tokyo, Japan), as an aqueous extract of the following 12 medicinal herbs (percentage): atractylodes rhizome (12.9), Japanese angelica root (12.9), *poria sclerotium* (12.9), *Rehmannia* root (12.9), ginseng (9.7), cinnamon bark (8.1), *citrus unshiu* peel (6.5), peony root (6.5), polygala root (6.5), astragalus root (4.8), *Glycyrrhiza* (3.2), and schisandra fruit (3.2). NYT formulations excluding each one of the 12 herbal crude drugs were also obtained from Kracie Pharma, Ltd. The dried powdered extract of NYT and its crude drugs were suspended in sterile water at 100 mg/ml concentration, diluted 100-fold with Hanks' balanced salt solution (in mM: 1.3 CaCl_2_·2*H*_2_O, 0.81 MgSO_4_, 5.4 KCl, 0.44 KH_2_PO_4_, 4.2 NaHCO_3_, 136.9 NaCl, 0.34 Na_2_HPO_4_, and 5.6 D-glucose) containing 20 mM HEPES and 0.1% BSA, and filtered through a 0.2 μm membrane (KURABO Industry Ltd., Osaka, Japan). The solution was used to treat cells at final concentrations of 3, 10, 30, and 100 μg/ml. All other reagents were of the highest purity available from commercial sources.

### Generation of Stable Cell Lines

GHSR-expressing cells were cultured as described previously (33). The expression vector C-terminal FLAG-tagged human GHS-R1a was transfected into human embryonic kidney 293A (HEK293A) cells using PEI Max (Polysciences, Inc., Warrington, PA, USA). For human NPY1R and NPY5R clones, we synthesized the combined fragment of the NruI restriction site, human EF1 promoter (34), N-terminal cleavable hemagglutinin secretion signal (MKTIIALSYIFCLVFA) (35), and NheI site and inserted it into the pIRESpuro3 expression vector (Takara, Shiga, Japan) using its recognition site (pEF1-IRESpuro). Subsequently, we amplified the cDNA encoding hNPY1R (NM_000909.6) ORF and hNPY5R (NM_001317091.1) ORF with the primers tagged with the NheI (N′) and BamHI (C′) sites from a full-length cDNA clone (Genscript, Piscataway, NJ, USA), and it was transferred into the pEF1-IRESpuro vector. The expression constructs were transfected into HEK293T cells according to the manufacturers' instructions, and 48 h after transfection, cells stably expressing either NPY1R or NPY5R were selected. The human OX1R clone (GenBank accession: AB463762; Kazusa DNA Research Institute, Chiba, Japan) was amplified according to the manufacturer's instructions. HEK293 cells (American Type Culture Collection, Manassas, VA, USA) stably expressing OX1R were generated through transfection of plasmids using ScreenFect™ (Fujifilm Wako Pure Chemical) and selected based on the OX1R activity measured using the CellKey™ assay. Ethical approval of the experimental procedures was obtained from National Cancer Center Research Institute (approval no. B85M1-13).

### Cell Culture

All cells were cultured at 37°C in a humidified atmosphere of 95% air and 5% CO_2_. GHSR-expressing HEK293A cells and OX1R-expressing HEK293 cells were maintained in DMEM (Gibco or Fujifilm Wako Pure Chemical) supplemented with 10% FBS, penicillin (100 U/ml), streptomycin (100 mg/ml), and geneticin (800 μg/ml). HEK293T cells expressing NPY1R or NPY5R were maintained in DMEM (Gibco) supplemented with sodium pyruvate (1 mM), 10% FBS, penicillin (100 U/ml), and streptomycin (100 mg/ml).

### Measurement of GPCR Activity Using the CellKey™ System

The assay with the CellKey™ system was conducted as described previously (36–40). Briefly, the cells were cultured at a density of 4.0 × 10^4^ (GHSR-expressing HEK293A), 6.0 × 10^4^ (NPY1R-expressing HEK293T), 5.0 × 10^4^ (NPY5R-expressing HEK293T), and 6.0 × 10^4^ cells/well (OX1R-expressing HEK293) in CellKey™ 96-well microplates. After incubating at 37°C for 24 h, the cells were washed with Hanks' balanced salt solution containing 20 mM HEPES and 0.1% BSA, and allowed to equilibrate in the assay buffer for 30 min before the assay. The CellKey™ instrument applies small voltages to the electrodes every 10 s and measures impedance of the cell layer. In this study, we recorded at 5 min baseline, added drugs, and measured changes in impedance (ΔZ) for 25 min. The rate of change in impedance is expressed as the difference of the minimum impedance and maximum impedance after drug injection as previously reported (39).

### Statistical Analysis

The data are presented as mean ± S.E.M. The statistical analyses were performed using the one-way analysis of variance (ANOVA) followed by Bonferroni's multiple comparison test (GraphPad Prism 8, GraphPad Software, San Diego, CA, USA). The results with a probability value *p* < 0.05 were considered statistically significant.

## Results

### NYT Activated OX1R but Not GHSR, NPY1R, and NPY5R

**Figure 1:**
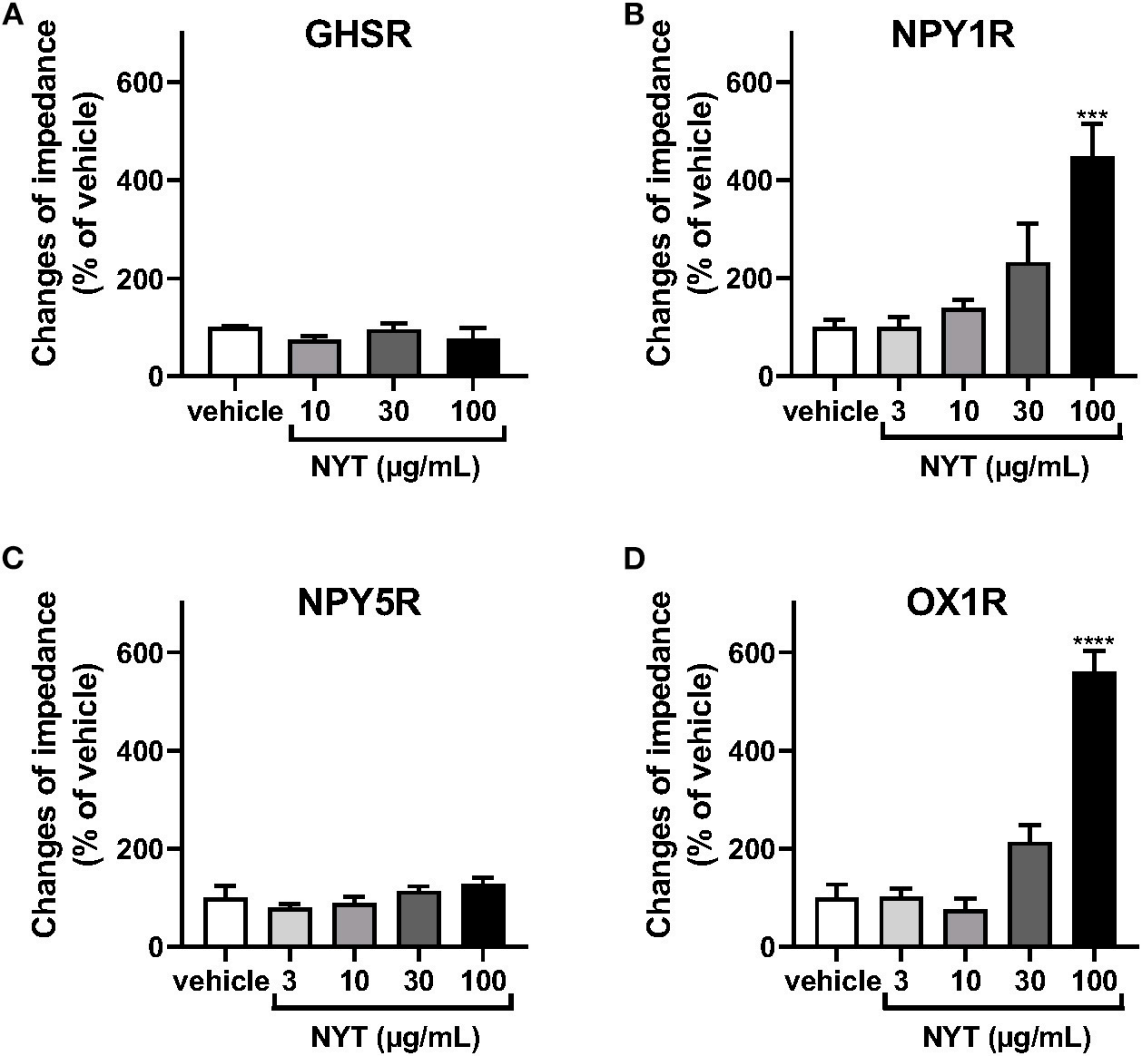
Effects of Ninjinyoeito (NYT) on impedance changes in cells expressing several G-protein-coupled receptors, which activate hyperphagia signaling in the central nervous system using the CellKey™ assay. The cells stably expressing growth hormone secretagogue receptor 1a (GHSR) (**A**, *n* = 6–8), neuropeptide Y1 receptor (NPY1R) (**B**, *n* = 6), neuropeptide Y5 receptor (NPY5R) (**C**, *n* = 6), or orexin 1 receptor (OX1R) (**D**, *n* = 6) were treated with NYT (3–100 μg/kg) or its vehicle (control). The rate of change in impedance was measured using the CellKey™ system and expressed as the difference of the minimum impedance and maximum impedance after drug injection. The data are expressed as mean ± S.E.M. *** and **** indicate *p* < 0.001 and *p* < 0.0001, respectively, compared with the control; Bonferroni's multiple comparison test following one-way ANOVA.

**Figure 2:**
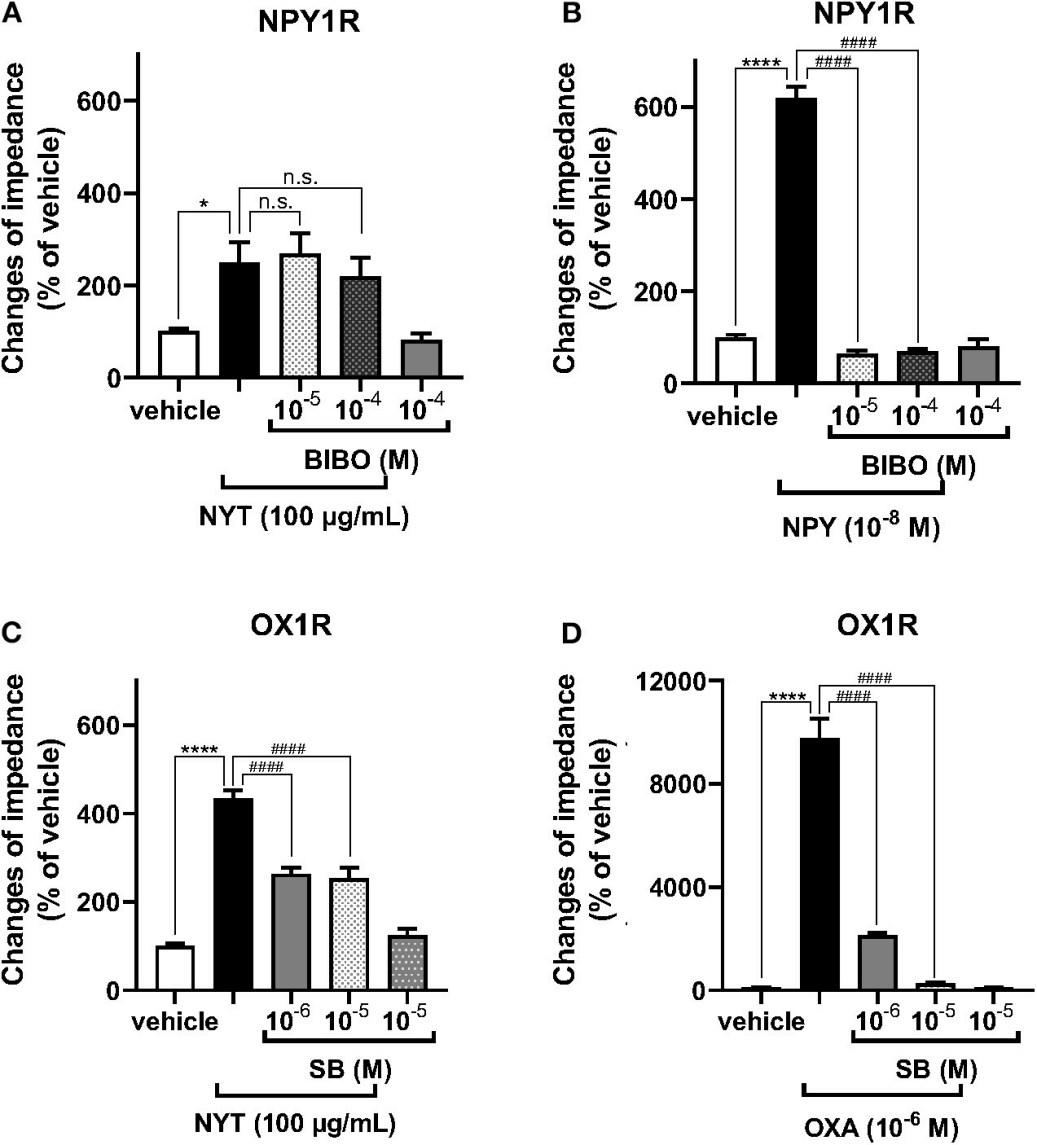
Effects of the antagonists of NPY1R and OX1R on the NYT-induced increase in impedance of cells expressing NPY1R or OX1R. The cells expressing NPY1R **(A,B)** were pretreated with or without BIBO3340 (BIBO, 10^−5^ or 10^−4^ M), a selective NPY1R antagonist, for 30 min and then treated with NYT (**A**, 100 μg/ml) or NPY (**B**, 10^−8^ M), respectively. After treatment with the vehicle or selective OX1R antagonist SB676042 (SB, 10^−6^ or 10^−5^ M) for 30 min, OX1R-expressing HEK293 cells were treated with NYT **(C)** or orexin A (**D**, OXA). The rate of change in impedance is expressed as the difference of the minimum and maximum impedance after drug injection. The data are expressed as mean ± S.E.M. (*n* = 6–12). *p < 0.05, and ****p < 0.0001, respectively, compared with the vehicle (control); ####*p* < 0.0001, compared with NYT or each selective agonist; Bonferroni's multiple comparison test following one-way ANOVA.

We examined the effects of NYT on the activation of GHSR, NPY1R, NPY5R, and OX1R using the CellKey™ system. As shown in Figures 1A,C, NYT (3–100 μg/ml) did not significantly change the ΔZ of GHSR-expressing HEK293A cells and NPY5R-expressing HEK293T cells. On the contrary, the GHSR agonist ghrelin (10^−7^ M) and NPYR agonist neuropeptide Y (NPY, 10^−6^ M) increased the ΔZ of these cells, respectively, in GHSR-expressing cells: control vs. ghrelin (10^−7^ M) (% of control, mean ± S.E.M.), 100 ± 7.81 vs. 2,227.5 ± 288.4; in NPY5R-expressing cells: control vs. NPY (10^−6^ M), 100 ± 10.24 vs. 172.8 ± 9.68. NYT significantly increased the ΔZ of cells expressing NPY1R or OX1R in a dose-dependent manner (Figures 1B–D). However, the NYT-induced increase in ΔZ of NPY1R-expressing HEK293T cells was not significantly attenuated by BIBO3340 (BIBO, 10^−5^ or 10^−4^ M), at concentrations that completely inhibited the increase in ΔZ induced by NPY (10^−8^ M) (Figures 2A,B). Contrarily, SB676042 (SB, 10^−6^ or 10^−5^ M) significantly suppressed the NYT-induced increase in ΔZ of OX1R-expressing HEK293 cells (Figure 2C). Pretreatment with SB676042 (SB, 10^−6^, or 10^−5^ M) significantly inhibited the increase in ΔZ induced by the OX1R agonist orexin A (OXA) in a dose-dependent manner (Figure 2D).

### Only Citrus Unshiu Peel, One of the Herbal Crude Drugs in NYT, Elicited OX1R Activation

**Figure 3:**
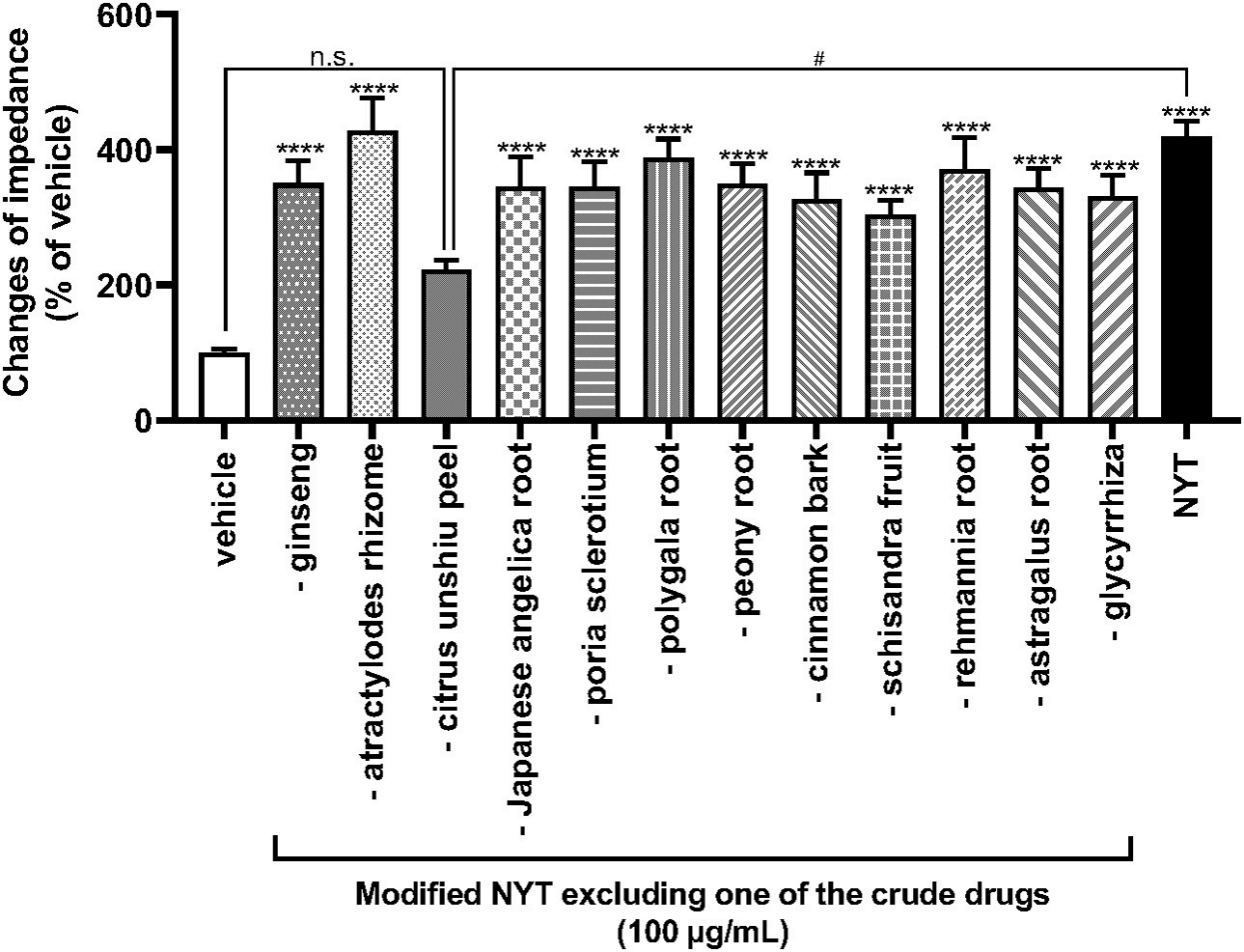
Effects of modified NYT excluding each one of the 12 herbal crude drugs on the increase in impedance of OX1R-expressing HEK293 cells. HEK293 cells stably expressing OX1R were treated with modified NYT excluding 1 herbal crude drug (100 μg/ml) from 12 ingredients of NYT or its vehicle (control). The rate of change in impedance is expressed as the difference of the minimum and maximum impedance after drug injection. The data are expressed as mean ± S.E.M. (*n* = 6). ****p < 0.0001, compared with the control; #*p* < 0.05, compared with the NYT; Bonferroni's multiple comparison test following one-way ANOVA.

To clarify the medical herbal crude drugs involved in NYT-induced OX1R activation, we investigated the effects of modified NYT excluding 1 of the 12 herbal drugs composing NYT on the increase in ΔZ of OX1R-expressing HEK293 cells. As shown in Figure 3, only the modified NYT without *citrus unshiu* peel (100 μg/ml) failed to significantly increase the ΔZ of OX1R-expressing cells; compared with the NPY-induced increase in ΔZ, the responses were significantly low (Figure 3).

**Figure 4:**
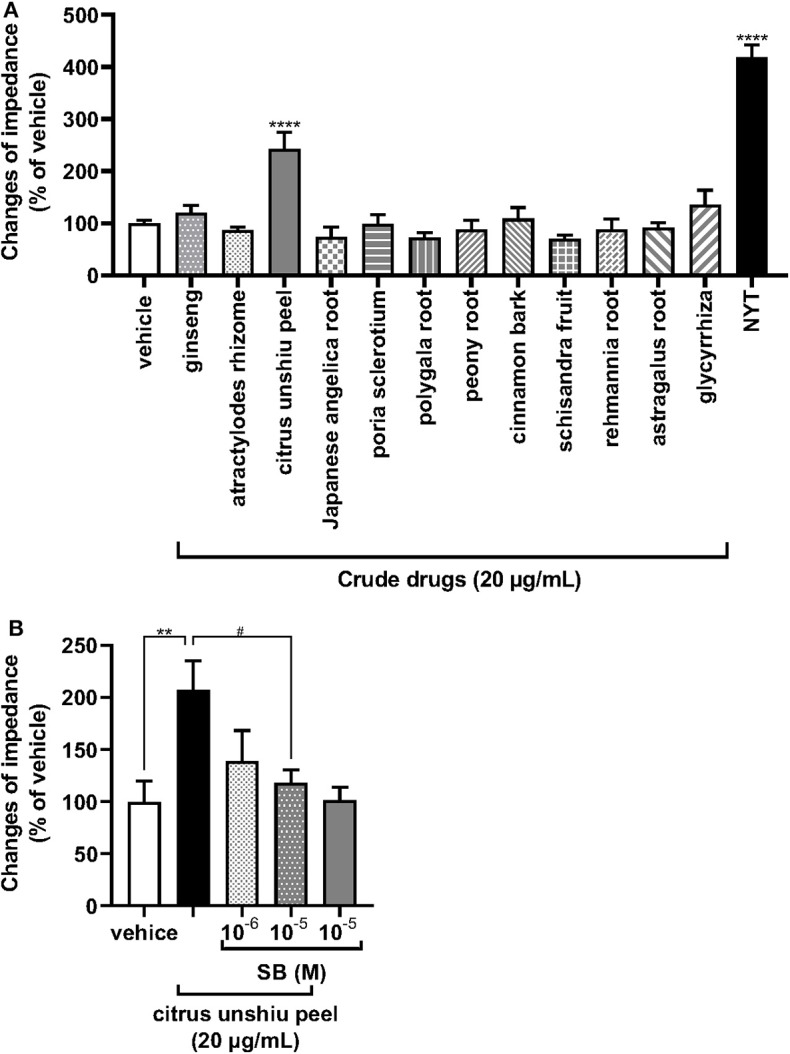
Effects of each herbal crude drug containing NYT on the increase in impedance of OX1R-expressing HEK293 cells. **(A)** HEK293 cells stably expressing OX1R were treated with each herbal crude drug alone contained in NYT (20 μg/ml) or its vehicle (control) (*n* = 6–12). **(B)** The cells expressing OX1R were pretreated with or without the selective OX1R antagonist SB676042 (SB, 10^−6^ or 10^−5^ M) for 30 min and then treated with *citrus unshiu* peel (20 μg/ml) (*n* = 5–6). The rate of change in impedance is expressed as the difference of the minimum and maximum impedance after drug injection. The data are expressed as mean ± S.E.M (*n* = 6). ** < 0.001, and ****p < 0.0001, respectively, compared with the vehicle (control); #*p* < 0.05, compared with *citrus unshiu* peel; Bonferroni's multiple comparison test following one-way ANOVA.

We further examined the effects of each of the medical herbal crude drugs composing NYT on the activity of OX1R. NYT contains 12 medicinal herbs in the following ratio: atractylodes rhizome (4), Japanese angelica root (4), *poria sclerotium* (4), *Rehmannia* root (4), ginseng (3), cinnamon bark (2.5), *citrus unshiu* peel (2), peony root (2), polygala root (2), astragalus root (1.5), *Glycyrrhiza* (1), and schisandra fruit (1). Thus, the major components of the herbal drugs were atractylodes rhizome, Japanese angelica root, poria sclerotium, and *Rehmannia* root, each accounting for 13% of NYT. We therefore analyzed the effects of 20 μg/ml of the 12 individual medicinal herbs (20% of the volume of NYT) on the activity of OX1R to reveal the medicinal herbs in NYT that activate OX1R. As shown in Figure 4A, only *citrus unshiu* peel (20 μg/ml) significantly increased the ΔZ (Figure 4A), and the responses were suppressed by pretreatment with SB676042 (SB, 10^−6^ and 10^−5^ M) in a dose-dependent manner (Figure 4B).

## Discussion

The present study, to the best of our knowledge, for the first time, revealed that NYT activated OX1R but not GHSR, NPY1R, and NPY5R, which are receptors of various hypothalamic peptides regulating feeding behavior. Moreover, we found that *citrus unshiu* peel in NYT induced the activation of OX1R. These data suggest that *citrus unshiu* peel in NYT is an activator of feeding behavior.

NPY is a 36-amino-acid neuropeptide and abundantly distributed in the arcuate nucleus of the hypothalamus, which integrates signals for energy homeostasis (18). NPYR is classified into five subtypes (NPY1, NPY2, NPY3, NPY5, and NPY6), and NPY1R and NPY5R play roles in appetite control (30). The present results showed that NYT induced an increase of ΔZ in HEK293T cells expressing NPY1R but not NPY5R (Figure 1). However, the selective NPY1R antagonist BIBO3340 did not significantly decrease NPY1R-induced responses (Figure 2A). Recently, it has been reported that NPYR forms heterodimers with other GPCRs (41, 42). In some cases, the responses of GPCR heterodimers were not notably inhibited by the antagonist of each GPCR monomer (43). Overall, our present data suggest that NYT might activate NPY1R/endogenous unidentified GPCRs expressed in HEK293 cells as heterodimers.

Orexin (orexin A and orexin B), one of the neuropeptides, was initially recognized as a regulator of feeding behavior, because of its exclusive production in the lateral hypothalamic area (LHA), a region known as the feeding center (17, 20, 29, 31). The orexin receptor is classified into two subtypes (OX1R and OX2R), and OX1R is mainly involved in the increase in food intake (20, 22, 31). In this study, NYT-induced OX1R activities were significantly attenuated by SB676042 (Figure 2C), suggesting that NYT could be an agonist of OX1R. Modified NYT without *citrus unshiu* peel (100 μg/ml) failed to increase OX1R activities in the cells (Figure 3), and the increase was completely suppressed by SB676042 (Figure 4). However, the level of increase in ΔZ induced by NYT or *citrus unshiu* peel [NYT (% of control): 433.3 ± 19.5, *citrus unshiu* peel: 207.4 ± 28.0] was considerably smaller than that caused by OXA (% of control: 9,781.5 ± 252.9). Theoretically, kampo medicine comprises several medical herbal crude drugs; thus, it is considered to exert multiple actions (11, 44–51). Furthermore, each action of kampo medicine is considered milder than that of a drug composed of only one component, such as western drugs. Thus, kampo medicines are known to cause fewer adverse effects (52). Taken together, these results suggest that NYT might have other actions, besides the activation of OX1R, to improve appetite and fatigue.

In the present study, modified NYT excluding *citrus unshiu* peel (100 μg/ml) did not significantly activate OX1R (Figure 3). In addition, the OX1R activities were induced by *citrus unshiu* peel alone, and this was suppressed by SB676042 (Figure 4). These data suggest that *citrus unshiu* peel is an agonist of OX1R. In an aqueous extract mixture of NYT, the main ingredients of *citrus unshiu* peel were hesperidine, nobiletin, tangeretin, heptamethoxyflavone, naringin, and synephrine (53–55). Some studies on blood pharmacokinetics indicated that these ingredients are absorbed into the blood in humans after oral administration (56, 57). In addition, some reports have shown permeation of polymethoxyflavones and nobiletin into the brain using animal models (58, 59). Therefore, these data suggest that ingredients derived from *citrus unshiu* peel could pass the blood–brain barrier (BBB), and reach the OX1R on neurons. The facts suggest that these ingredients in *citrus unshiu* peel could pass the BBB and act as agonists of OX1R in neurons. However, further studies are required, and we will elucidate the ingredients that induce the activation of OX1R in the future.

Several studies have shown that cancer cachexia induced a decrease in food intake in accordance with the changes in orexigenic/anorexigenic neuropeptides such as orexin. This suggests that modulating orexigenic/anorexigenic neuropeptide expression is important for patients with cancer to improve cachexia (10, 18). In this study, we revealed that NYT and *citrus unshiu* peel activated OX1R using an *in vitro* assay. Kim et al. have shown that *citrus unshiu* peel extract alleviates cancer-induced weight loss in mice bearing CT-26 adenocarcinoma (60). Although further studies using cancer cachexia–anorexia model animals are required, these previous and our present data suggest that NYT might improve cancer cachexia–anorexia via the activation of OX1R.

Ghrelin, a 28-amino-acid peptide, is mainly secreted from X/A-like cells in the stomach as an orexigenic peptide (19, 28, 32). The ghrelin receptor GHSR is primarily located in NPY and agouti-related protein (AGRP) containing neurons of the hypothalamus–pituitary unit (21–28). The plasma ghrelin levels increase in response to prolonged fasting and rapidly decrease after feeding, suggesting that peripheral ghrelin is significantly important for appetite regulation (26). We previously reported that rikkunshito (RKT), a Japanese herbal kampo medicine, improved appetite as assessed using a visual analog scale (VAS) in a randomized phase II study (61). In addition, we previously revealed the mechanism through which RKT ameliorated anorexia in cancer cachexia model rats (32, 62). It has been demonstrated that RKT alleviated ghrelin resistance by enhancement of ghrelin signaling (32). We also previously reported that atractylodin, an ingredient in *Atractylodes lancea* rhizome, an herbal drug composing RKT, enhanced ghrelin-induced GHSR activation via an increase in the ghrelin/GHSR-binding activity (33). However, NYT does not contain *Atractylodes lancea* rhizome, and the present study showed that NYT neither activated GHSR (Figure 1A) nor enhanced the ghrelin-induced GHSR activation (Supplementary Figure 1). These data suggest that although both RKT and NYT are involved in the improvement of anorexia, they may ameliorate cancer cachexia–related anorexia via different mechanisms.

In conclusion, the present results suggest that NYT and its ingredient *citrus unshiu* peel activated OX1R. Although further studies using animal models with cancer cachexia–anorexia are needed, these data suggest that NYT might improve cancer cachexia–anorexia partially via activation of OX1R. This study provides scientific evidence supporting the use of NYT in patients with cancer cachexia–anorexia.

## Data Availability Statement

All datasets generated for this study are included in the article/Supplementary Material.

## Author Contributions

KM and YU: conceptualization and writing—review and editing. KM, KO, and YY: methodology: MN: validation. KO, NS, and SF: investigation. KM, KO, and NS: data curation. KM: writing—original draft preparation. YH, KY, and HF: supervision. YU: project administration and funding acquisition.

### Conflict of Interest

YU received grant support from Kracie Pharma, Ltd. The remaining authors declare that the research was conducted in the absence of any commercial or financial relationships that could be construed as a potential conflict of interest.
