# Supplementary material for: Japanese Herbal Medicine Ninjinyoeito Mediates Its Orexigenic Properties Partially by Activating Orexin 1 Receptors
Source: Front Nutr. 2020 Feb 27;7:5. doi: 10.3389/fnut.2020.00005 (PMC7056666; doi:10.3389/fnut.2020.00005)
Supplement: Supplementary file 1 [file Data_Sheet_1.pdf]

Methods

Measurement of GHSR activity using the CellKey™ system

The assay with the CellKey™ system was conducted as described previously (Miyano et al., 2014; Hisaoka-Nakashima et al., 2015; Kajitani et al., 2016; Meguro et al., 2018; Manabe et al., 2019). Briefly, GHSR-expressing HEK293A were cultured at a density of 4.0×10^4 cells/well in CellKey™ 96-well microplates. After incubating at 37°C for 24 h, the cells were washed with Hanks' balanced salt solution containing 20 mM HEPES and 0.1% BSA and allowed to equilibrate in the assay buffer for 30 min before the assay. The CellKey™ instrument applies small voltages to the electrodes every 10 s and measures impedance of the cell layer. In this study, we recorded at 5-min baseline, added drugs, and measured changes in impedance for 25 min. The rate of change in impedance is expressed as the difference of minimum impedance and maximum impedance after drug injection as previously reported (Meguro et al., 2018).

Results

NYT did not enhance the ghrelin-induced increase in impedance

We examined the effects of NYT on the ghrelin-induced increase in impedance using CellKey system. Compared with vehicle (ghrelin alone), 100 μg/mL NYT did not significantly enhanced ghrelin-induced increase in impedance (**Supplementary Figure. 1**). These data suggest that NYT did not enhance the ghrelin-induced GHSR activation.

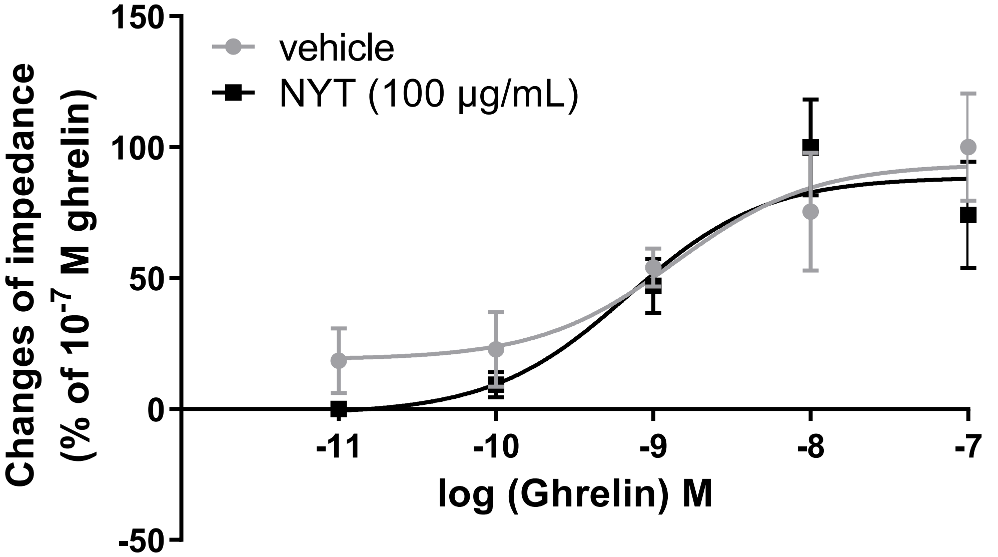

Supplementary Figure. 1 Effects of NYT on the ghrelin-induced increase in impedance in GHSR-expressing HEK293A cells

The cells were pretreated with vehicle or 100 µg/mL NYT for 30 min, and treated with ghrelin (10^{-11}-10^{-7} M) for 25 min. The data were expressed as the mean ± S.E.M., and analyzed by Bonferroni's multiple comparisons test following two-way ANOVA.
